# The Mechanisms Responsible for Improved Information Transfer in Avatar-Based Patient Monitoring: Multicenter Comparative Eye-Tracking Study

**DOI:** 10.2196/15070

**Published:** 2020-03-16

**Authors:** David Werner Tscholl, Julian Rössler, Lucas Handschin, Burkhardt Seifert, Donat R Spahn, Christoph B Nöthiger

**Affiliations:** 1 Institute of Anesthesiology University Hospital Zurich Zurich Switzerland; 2 Department of Biostatistics Epidemiology, Biostatistics and Prevention Institute University of Zurich Zurich Switzerland

**Keywords:** computers, diagnosis, visual perception, awareness, patient safety

## Abstract

**Background:**

Patient monitoring is central to perioperative and intensive care patient safety. Current state-of-the-art monitors display vital signs as numbers and waveforms. Visual Patient technology creates an easy-to-interpret virtual patient avatar model that displays vital sign information as it would look in a real-life patient (eg, avatar changes skin color from healthy to cyanotic depending on oxygen saturation). In previous studies, anesthesia providers using Visual Patient perceived more vital signs during short glances than with conventional monitoring.

**Objective:**

We aimed to study the deeper mechanisms underlying information perception in conventional and avatar-based monitoring.

**Methods:**

In this prospective, multicenter study with a within-subject design, we showed 32 anesthesia providers four 3- and 10-second monitoring scenarios alternatingly as either routine conventional or avatar-based in random sequence. All participants observed the same scenarios with both technologies and reported the vital sign status after each scenario. Using eye-tracking, we evaluated which vital signs the participants had visually fixated (ie, could have potentially read and perceived) during a scenario. We compared the frequencies and durations of participants’ visual fixations of vital signs between the two technologies.

**Results:**

Participants visually fixated more vital signs per scenario in avatar-based monitoring (median 10, IQR 9-11 versus median 6, IQR 4-8, *P*<.001; median of differences=3, 95% CI 3-4). In multivariable linear regression, monitoring technology (conventional versus avatar-based monitoring, difference=−3.3, *P*<.001) was an independent predictor of the number of visually fixated vital signs. The difference was less prominent in the longer (10-second) scenarios (difference=−1.5, *P*=.04). Study center, profession, gender, and scenario order did not influence the differences between methods. In all four scenarios, the participants visually fixated 9 of 11 vital signs statistically significantly longer using the avatar (all *P*<.001). Four critical vital signs (pulse rate, blood pressure, oxygen saturation, and respiratory rate) were visible almost the entire time of a scenario with the avatar; these were only visible for fractions of the observations with conventional monitoring. Visual fixation of a certain vital sign was associated with the correct perception of that vital sign in both technologies (avatar: phi coefficient=0.358; conventional monitoring: phi coefficient=0.515, both *P*<.001).

**Conclusions:**

This eye-tracking study uncovered that the way the avatar-based technology integrates the vital sign information into a virtual patient model enabled parallel perception of multiple vital signs and was responsible for the improved information transfer. For example, a single look at the avatar’s body can provide information about: pulse rate (pulsation frequency), blood pressure (pulsation intensity), oxygen saturation (skin color), neuromuscular relaxation (extremities limp or stiff), and body temperature (heatwaves or ice crystals). This study adds a new and higher level of empirical evidence about why avatar-based monitoring improves vital sign perception compared with conventional monitoring.

## Introduction

The World Health Organization considers continuous patient monitoring to be “extremely important” for the safety of the more than 313 million patients undergoing surgery worldwide each year [1,2]. In operating rooms and intensive care units around the world, monitors help millions of health care providers make critical treatment decisions [3,4]. However, previous research has found that conventional patient monitoring based on numbers and waveforms is not ideally suited for transferring patient status information to health care providers [5-7]. These studies recommend the development of new technologies to improve information transfer, especially from short glances at the monitors, because that is how care providers perform monitoring in real life.

In a previous comparative study with conventional monitoring, we found that anesthesia professionals were able to perceive more vital signs when monitoring with Visual Patient, a technology integrating vital sign information into an easy-to-interpret animated avatar model of the patient’s status, designed according to principles of user-centered design [4,8,9]. When using avatar-based monitoring, participants rated their self-confidence in the correctness of their diagnoses as higher and their subjectively perceived workload as lower.

Although the biocular human visual field encompasses approximately 214 arc degrees horizontally and 150 arc degrees vertically, we can only see sharply in a circular area of approximately 2 arc degrees in the center of our visual field, named the fovea [10]. While reading, we move our eyes to let the light reflected from the words fall through the pupil and the lens directly onto the fovea. At a distance of approximately one arm’s length, the foveal region in which we can see sharp, colorful images is approximately the size of a thumbnail or a circle with a radius of 2 centimeters [11,12].

Human eye movements take place in the form of so-called fixations and saccades. Visual fixations are the periods during which the gaze rests on a position, and information can reach the visual cortex and potentially be interpreted. Saccades are the rapid movements of the eyeballs between fixations [13]. Eye-tracking technology can record both visual fixations and saccades. For this study, we systematically recorded, analyzed, and compared eye-tracking data of participants who watched patient-monitoring scenarios alternatingly as conventional and avatar-based patient monitoring. The rationale of this study was to uncover the underlying functional principles in both monitoring technologies through eye-tracking analysis. These results may be useful for improved understanding of avatar-based monitoring and, across domains, for the future development of user interfaces designed to transfer relevant information as efficiently as possible. We hypothesize that avatar-based monitoring (Visual Patient) facilitates information perception through its compact layout, which enables users to visually fixate on more vital signs.

## Methods

In this paper, we describe the analysis of eye-tracking data that was collected as part of a multimethod laboratory study. The primary objective of that study was to compare the perceptual performance of anesthesia professionals using newly developed avatar-based technology with state-of-the-art number- and waveform-based patient monitoring [8].

The Ethics Committee of the Canton of Zurich, Switzerland, reviewed the study protocol and issued a declaration of nonresponsibility specifying that the research project did not fall into the scope of the Human Research Act (Business Administration System for Ethics Committees, number: 2016-00103). Nevertheless, we obtained written consent for the use of the collected data for scientific purposes from all participants.

### Description of Visual Patient Technology

**Figure 1:**
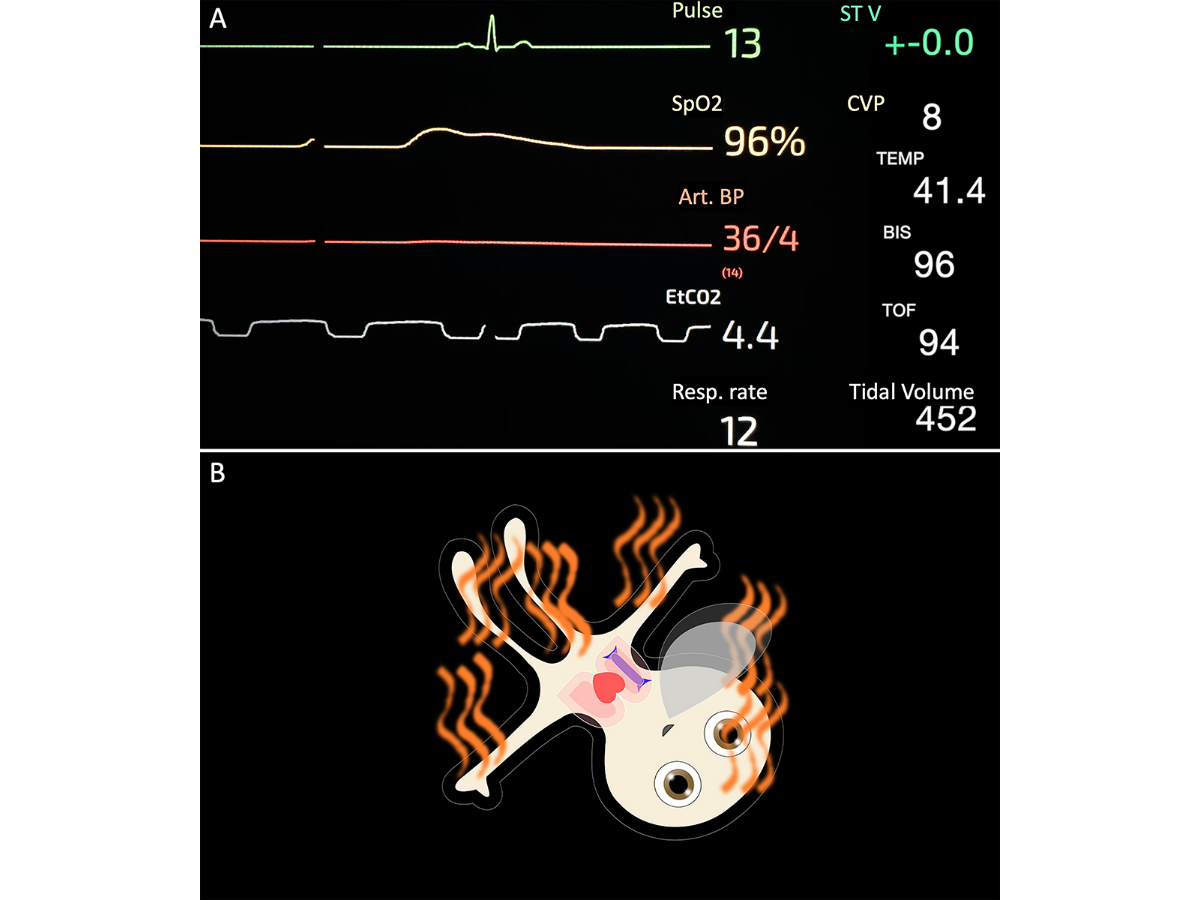
Patient monitoring scenario used in this study: (A) presented in routine conventional number and waveform-based format; (B) presented in avatar-based format (Visual Patient). SpO_2_: Peripheral oxygen saturation; Art BP: arterial blood pressure; EtCO_2_: end-tidal carbon dioxide concentration; Resp rate: respiratory rate; ST V: electrocardiogram ST segment of lead number 5; CVP: central venous pressure; Temp: body temperature; BIS: bispectral index system (brain activity); TOF: train of four (neuromuscular relaxation).

Visual Patient, as used in this study, can display the 11 most frequently monitored vital signs: pulse rate, blood pressure, oxygen saturation, ST segment of the electrocardiogram, central venous pressure, respiratory rate, tidal volume, expiratory carbon dioxide concentration, body temperature, brain activity, and degree of neuromuscular relaxation. We developed the technology as a situation awareness tool, analogous to synthetic vision technology in aviation, according to the principles of user-centered design and principles of logic [4,14]. Synthetic vision technology generates a virtual image of the environment from the data measured by the aircraft (eg, airspeed) and Global Positioning System geolocation data and data stored on onboard computers (eg, georeferenced terrain elevation data). For the pilot, the generated virtual image looks like the view from the cockpit in perfect weather conditions. This similarity between the virtual image and reality makes the image intuitively understandable and enables a quick and uncomplicated perception of the flight situation. Visual Patient uses the same logic by creating a virtual image of the patient from vital data. It presents the data in a way that corresponds to the real phenomena that the data would cause in the patient. For example, the pulse rate corresponds to the pulsation of the avatar’s body to represent the pulse wave passing through the body with every heartbeat. High brain activity is represented by open eyes because that is what the care providers expect from a patient with high brain activity according to their mental models. Figure 1 shows a monitoring scenario in routine conventional form and as a Visual Patient representation.

This direct presentation of information eliminates the need to calculate the relevant information (eg, “What is the current anesthesia depth?”) from lower-level data (eg, bispectral index=85) [15]. In addition to this direct presentation of information, the other two main features of the avatar technology are the preprocessing of data for each vital sign into categories (“no data measured,” “too low,” “normal,” or “too high”) and the presentation of vital parameter information in multiple visualizations simultaneously. For example, the caregiver can evaluate the respiratory rate based on the respiratory rate of the avatar lung and the formation rate of the carbon dioxide cloud exhaled by the avatar.

These combined functions translate a large number of numerical values into an animated model of the patient situation, which the caregiver can evaluate and memorize at a glance. The vital parameters are translated into the avatar model in real time from the monitoring data. If no data are measured for a particular vital sign, the corresponding visualization in the Visual Patient remains gray and framed with dashes. We have described the validation and evaluation process of the technology in detail in previous studies [8,16].

### Study Participants

The participants in this study were attending and resident physician anesthesiologists and specialist anesthesia nurses from the anesthesia departments of the University Hospital Zurich and the Cantonal Hospital of Winterthur. The University Hospital Zurich is one of the largest university hospitals in Switzerland, where more than 30,000 operations are performed per year; the Cantonal Hospital Winterthur is a large regional teaching hospital where approximately 10,000 operations are performed per year.

Participation in this study was voluntary, and there was no monetary compensation for the participants. We recruited colleagues who responded to an institutional invitation and recruited additional colleagues according to availability. At both centers, we included equal numbers of male and female participants and participants from three professional groups: (1) senior anesthesia physicians, (2) resident physicians, and (3) anesthesia nurses.

### Study Setting

Before data collection, the participants received training in avatar-based monitoring through a 6-minute educational video (Multimedia Appendix 1). The participants also familiarized themselves with the layout of the conventional monitoring used in the study: a simulation of a GE Datex Ohmeda Monitors (General Electric Company, Boston, MA, USA) recorded with the SimMon App (Castle 2 Andersen ApS, Hillerød, Denmark), which was equivalent to patient monitoring in routine use in the two centers. There was no additional training in conventional patient monitoring because all participants had at least one full year, some even decades, of anesthesia experience. The eye-tracking data were recorded during the evaluation of patient monitoring scenarios. In random order, we presented participants with 3- and 10-second prerecorded videos of patient monitoring scenarios shown in avatar and state-of-the-art number and waveform format. Multimedia Appendix 2 provides examples of a conventional and an avatar-based scenario. Each participant rated four videos in sequence. These videos consisted of a 3- and a 10-second monitoring scenario, each of which was shown twice, once with either technology. The scenarios came from a pool of four total scenarios, as outlined in Multimedia Appendix 3. The scenarios were designed with unambiguously safe or unsafe vital sign values and contained random vital sign abnormalities to avoid pattern recognition (ie, inferring the status of vital signs based on the status of the other vital signs).

To blind the participants to the fact that they were evaluating the same scenarios twice (once with either technology), we showed the scenarios in alternating order, starting with a random first scenario. Multimedia Appendix 3 shows a flowchart detailing this procedure. The scenario playback was performed on an Aspire V15 Nitro 15-inch laptop computer (ACER, Inc, Taipei, Taiwan) in ultra-high resolution (3840×2160 pixels) at 60 frames per second. The conventional monitoring scenarios included a standard audio signal with frequency and pitch for heart rate and oxygen saturation.

After brief time intervals, the screens darkened, and the participants indicated how they had perceived the 11 vital signs displayed in the scenarios as either “too low,” “too high,” “safe,” or “no recall.” We based this method on the Situation Awareness Global Assessment Tool developed by Endsley [4,17]. After each scenario, for every vital sign, the participants indicated how confident they felt that they had perceived it correctly. Furthermore, they were asked to rate their subjectively perceived workload for each scenario using the NASA (National Aeronautics and Space Administration) Task Load Index [18,19]. Data were collected using an iPad-based (Apple Inc, Cupertino, CA, USA) data collection tool [20].

### Recording and Analysis of Eye-Tracking Data

We evaluated the eye-tracking data for this study according to the physiological principles of the human eye and neurophysiological principles of human vision outlined in the Introduction. We used a stationary eye tracker (Gazepoint GP3, Gazept, Vancouver, BC, Canada) to capture visual fixations and saccades of participants observing conventional and avatar-based patient monitoring scenarios. The eye tracker recorded the position of the foveal vision on the screen 60 times per second and with 0.5 to 1 degree of visual angle accuracy.

### Outcome Measures

#### Vital Signs Fixated Per Scenario (Primary Outcome)

We chose to compare visual fixations in this study because we regarded them as a relevant requirement for perception. Based on the anatomic and physiologic principles outlined in the Introduction, we analyzed each visual fixation longer than 50 milliseconds in the eye-tracking recordings of each participant and scenario; for each visual fixation, we counted the vital signs that were within 2 centimeters of the fixation. Using this method, we identified the vital signs that participants could potentially have read during the recording because they were within the potentially readable visual area. A video demonstrating this method is available in Multimedia Appendix 2. Information can only reach the brain for processing after reading, which requires a visual fixation.

Conventional patient monitoring shows the vital signs on the screen in the form of numbers or waveforms. In conventional monitoring, if a participant had a visual fixation within 2 centimeters of the number or waveform representing a certain vital sign (eg, pulse rate), we counted a visual fixation for this vital sign. By comparing the numbers of vital sign visual fixations between the two technologies, we wanted to find out whether the participants could visually fixate on more vital signs with either one of the two technologies.

#### Visual Fixations Per Vital Sign

We also compared visual fixations for each of the 11 vital signs individually. This allowed us to determine whether vital signs were visually fixated more often with either one of the two monitoring technologies. We expected these findings to provide an explanation for the improved perceptive performance in avatar-based patient monitoring found in previous studies [8,16].

#### Duration of Visual Fixations Per Vital Sign

Analogous to the visual fixations per vital sign, we also compared the time durations of the visual fixations of each of the 11 vital signs with both monitoring technologies. In doing so, we evaluated whether either one of the two monitoring technologies would cause the vital signs to be visible for a longer time per observation. We analyzed this outcome measure because longer availability of the vital sign information could explain why participants’ perceptual performance was improved with avatar-based patient monitoring in previous studies [8,16].

#### Correlation of Vital Sign Visual Fixations With Correct Perception

To evaluate the association of visual fixation of a vital sign and its correct perception, we calculated phi correlation coefficients. If visual fixation correlated with correct perception, and the avatar enabled more vital signs to be seen per time interval, these results could validate both the study method and the avatar concept.

#### Correlation of Vital Sign Visual Fixations With Perceived Confidence

We calculated coefficients between visual fixation and diagnostic confidence to evaluate whether the visual fixation of a vital sign correlated with the subjectively perceived confidence in the correctness of one’s own diagnosis.

### Statistical Analysis

#### Sample Size Calculation

Before starting the study, we conducted a pilot study with five participants. We calculated the sample size using the effect size of 1.23 measured in the pilot study. Assuming a clinically relevant difference of one vital sign and an observed standard deviation of 0.81, the post hoc power analysis for a paired *t* test resulted in a sample size of eight participants, for an alpha error probability of 5% and a power of 0.8. To achieve this sample size in both centers and all four scenarios, we had to include at least 32 participants.

#### Descriptive Statistics and Normality Tests

Distribution of variables is expressed using medians and interquartile ranges (IQRs) regardless of normality. Normality was assessed with the Shapiro-Wilk test and visual inspection of quantile-quantile plots of dependent variables.

#### Univariate Statistics

Participants watched and evaluated the same monitoring scenarios with both monitoring technologies; therefore, we were able to perform intraparticipant comparisons. Depending on normality, we used either paired Student *t* tests or Wilcoxon signed rank tests to compare the number of vital sign visual fixations with both monitoring technologies. We calculated the 95% confidence interval (95% CI) of the median of differences using the Hodges-Lehmann estimate. To test the differences in visual fixations and duration of visual fixations per vital sign for statistical significance, we used Mann-Whitney tests. In this study, we performed multiple comparisons; therefore, we considered *P* values between .05 and .01 as trends and *P* values of <.01 as statistically significant.

#### Multivariable Linear Regression

Multivariable linear regression was performed with number of visually fixated vital signs between the monitoring technologies and its differences as dependent variables. Scenario duration, order of scenarios, center, profession, and gender of the participant served as possible predictors. Clustering of observations within the same participant was addressed using cluster robust standard errors.

#### Correlation Analyses

To test for associations between visual fixation of a vital sign and its correct perception as well as participants’ subjectively perceived confidence in the correctness of the diagnosis, we calculated chi-square tests for association and Pearson phi coefficients between visual fixation, accurate perception, and diagnostic certainty. The phi coefficient corresponds to a Pearson correlation coefficient estimated for two binary variables. We considered “very unconfident” and “unconfident” as 0 and “confident” and “very confident” as 1. If the frequency of an event was less than five, we used the Fisher exact test to assess statistical significance.

#### Statistical Software

We used Q*Power 3 (Heinrich-Heine-University, Düsseldorf, Germany) [21], Prism 8.1.1. (GraphPad Software, La Jolla, CA, USA), IBM SPSS Statistics 24 (International Business Machines Corporation, Armonk, NY, USA), and Stata 13.1 (StataCorp, College Station, TX, USA) for statistical analyses.

## Results

### Study and Participant Characteristics

Table 1 shows the characteristics of the study and the participants in detail. A total of 32 participants participated in the two study centers. Had we been able to record data from all 32 participants and scenarios, a theoretical maximum of 64 direct comparisons between avatar-based and conventional patient monitoring would have been possible. However, we were unable to record the eye-tracking data of two participants. For four other participants, we were only able to record one of two monitoring scenarios they watched due to technical problems. Despite the missing data, we were still able to evaluate 56 within-subject comparisons of eye-tracking data (88% of all 64 theoretically possible comparisons).

**Table 1:** Study and participant characteristics.

Characteristic			Study center		Total (N=32)
			University Hospital Zurich (n=16)	Cantonal Hospital Winterthur (n=16)	
Participants with successful eye-tracking recording, n			16	14	30
Direct comparisons, n			32	24	56
**Participants, n (%)**					
	Staff members		6 (37)	6 (37)	12 (37)
	Residents		4 (25)	4 (25)	8 (25)
	Nurse anesthetists		6 (37)	6 (37)	12 (37)
	**Gender, n (%)**				
		Female	7 (44)	10 (62)	17 (53)
		Male	9 (56)	6 (37)	15 (47)
	**Age group, n (%)**				
		25 to 34 years	10 (63)	6 (38)	16 (50)
		35 to 44 years	6 (38)	2 (13)	8 (25)
		45 to 54 years	0 (0)	6 (38)	6 (19)
		55 to 65 years	0 (0)	2 (13)	2 (6)
	**Anesthesia experience, n (%)**				
		<1 year	1 (6)	1 (6)	2 (6)
		1 to 5 years	5 (31)	4 (25)	9 (28)
		5 to 10 years	9 (56)	1 (6)	10 (31)
		>10 years	1 (6)	10 (63)	11 (34)
Monitors from different manufacturers previously used, median (IQR)			2 (2-3)	2 (1-4)	2 (1-3)
Duration of data collection session (minutes), median (IQR)			32 (28-35)	35 (32-41)	33 (30-39)
Duration of study (days), n			20	2	22

### Outcome Measures

#### Vital Signs Fixated Per Scenario

**Figure 2:**
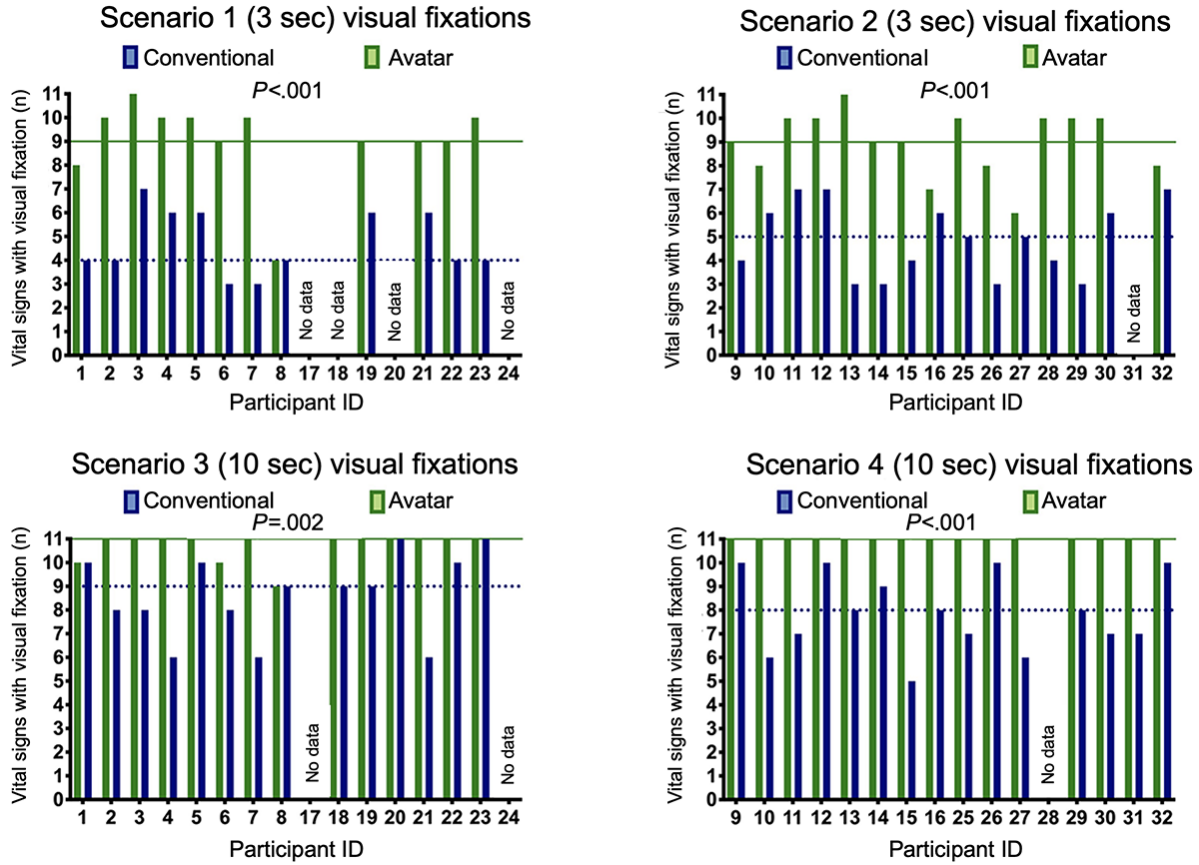
Avatar-based monitoring compared with conventional patient monitoring for vital signs visually fixated on by individual participants. Scenario 1 (3 seconds): n=12; scenario 2 (3 seconds): n=15; scenario 3 (10 seconds): n=14; and scenario 4 (10 seconds): n=15. The dotted lines indicate the medians. Participants 1-8 (University Hospital Zurich) and 17-24 (Cantonal Hospital Winterthur) rated scenarios 1 and 3; participants 9-16 (University Hospital Zurich) and 25-32 (Cantonal Hospital Winterthur) rated scenarios 2 and 4.

With the avatar-based monitoring, all participants in all scenarios were able to visually fixate on more vital signs than with conventional monitoring (Figure 2). In the short 3-second scenarios, the median numbers of vital sign fixations with avatar-based monitoring were approximately twice as high as conventional patient monitoring. In scenario 1, the avatar-based median was 9 (IQR 9-10) versus the conventional median of 4 (IQR 4-6, *P*<.001; median of differences=3, 95% CI 3-4). In scenario 2, the avatar median was 9 (IQR 8-10) versus the conventional median of 5 (IQR 3-6, *P*<.001; median of differences=5, 95% CI 2-6). In scenario 3, the first of the longer 10-second scenarios, the median number of vital sign fixations for avatar-based monitoring was 11 (IQR 11-11) versus the conventional median of 9 (IQR 6-10, *P*=.002; median of differences=2, 95% CI 0-4). In scenario 4, the second 10-second scenario, vital sign visual fixations were a median of 11 (IQR 11-11) for avatar versus the conventional median of 8 (IQR 7-10, *P*<.001; median of differences=3, 95% CI 1-4). Figure 1 shows these results on an individual participant level.

In a multivariable linear regression adjusted for scenario duration, order of scenarios, center, profession, and gender of the participant, the technology (conventional versus avatar-based monitoring) had a significant effect on the number of vital sign fixations: difference between technologies=−3.28, 95% CI −3.86 to −2.69, *P*<.001 (*F*_6,30_=145, Prob>*F*<.001, *R*^2^=.56, adjusted for clusters within participants). Table 2 shows the results of the multivariable linear regression for the difference of numbers of visually fixated vital signs between the technologies. In this analysis, only scenario duration affected the difference in vital sign fixations between technologies. The difference was less prominent in the 10-second scenarios (difference between scenario durations=−1.46, 95%CI −2.84 to −0.07, *P*=.04). Study center, profession, gender, and scenario order did not influence the differences between conventional and avatar-based monitoring.

**Table 2:** Multivariable linear regression for the difference in numbers of visually fixated vital signs between conventional and avatar-based monitoring.^a^

Variable	Difference (95% CI) (multivariable linear regression)	Standard error	Difference / standard error (multivariable linear regression)	*P* value
Scenario duration (3 versus 10 seconds)	−1.46 (−2.84 to −0.07)	0.68	−2.15	.04
Profession	0.33 (−0.87 to 1.52)	0.59	0.57	.58
Study center	−0.41 (−1.95 to 1.14)	0.76	−0.54	.60
Gender	0.01 (−1.20 to 1.22)	0.59	0.02	.99
Order of scenarios	0.10 (−1.51 to 1.70)	0.78	0.12	.90
Technology (conventional versus avatar [intercept])	−3.28 (−3.86 to −2.69)	0.29	−11.47	<.001

^a^ Clustering of observations within the same participant was addressed using cluster robust standard errors.

#### Visual Fixations Per Vital Sign

The analyses for each vital sign individually (Multimedia Appendix 3) showed that with avatar-based monitoring, 9 of 11 vital signs were fixated statistically significantly more often per scenario than with conventional patient monitoring. The vital signs for pulse rate, oxygen saturation, and blood pressure were visible in almost every fixation of participants in all four scenarios with avatar-based monitoring. In comparison, with conventional monitoring, each vital sign was readable only during a small number of visual fixations per observation.

#### Duration of Visual Fixations Per Vital Sign

**Figure 3:**
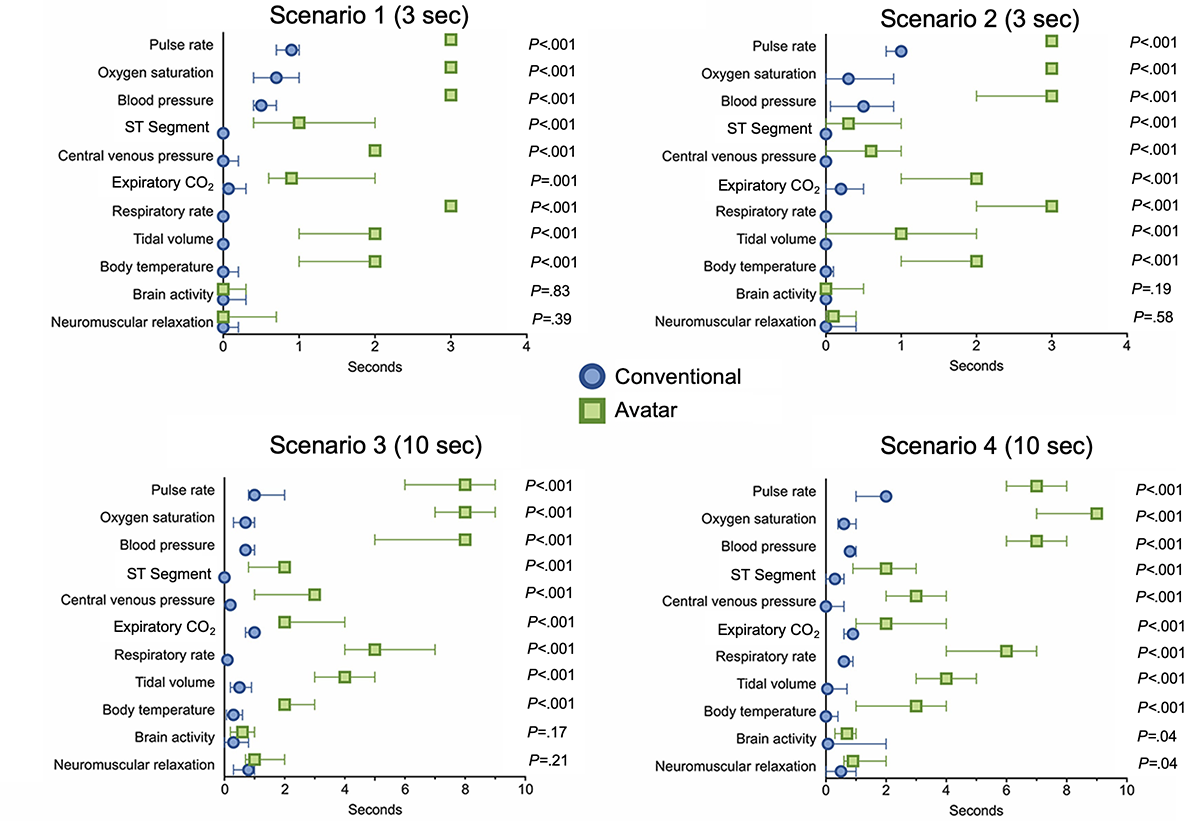
Avatar-based monitoring compared with conventional patient monitoring: median (with interquartile range) durations of visual fixations for each vital sign, scenario, and technology. Scenario 1 (3 seconds): n=12; scenario 2 (3 seconds): n=15; scenario 3 (10 seconds): n=14; and scenario 4 (10 seconds): n=15.

Similar to the number of fixations per vital sign, in all four scenarios, 9 of 11 vital signs were visually fixated significantly longer with the avatar than with conventional patient monitoring (Figure 3). With avatar-based monitoring, four critical vital signs (pulse rate, blood pressure, oxygen saturation, and respiratory rate) were visible to users during almost the entire time of the scenarios. This was because, in the avatar’s design, this information is displayed in the form of large anatomical objects, which extend across large parts of the screen. For example, the body of the avatar and the exhaled CO_2_ cloud (Figure 1).

#### Correlation of Vital Sign Visual Fixations With Correct Perception and Perceived Confidence

**Figure 4:**
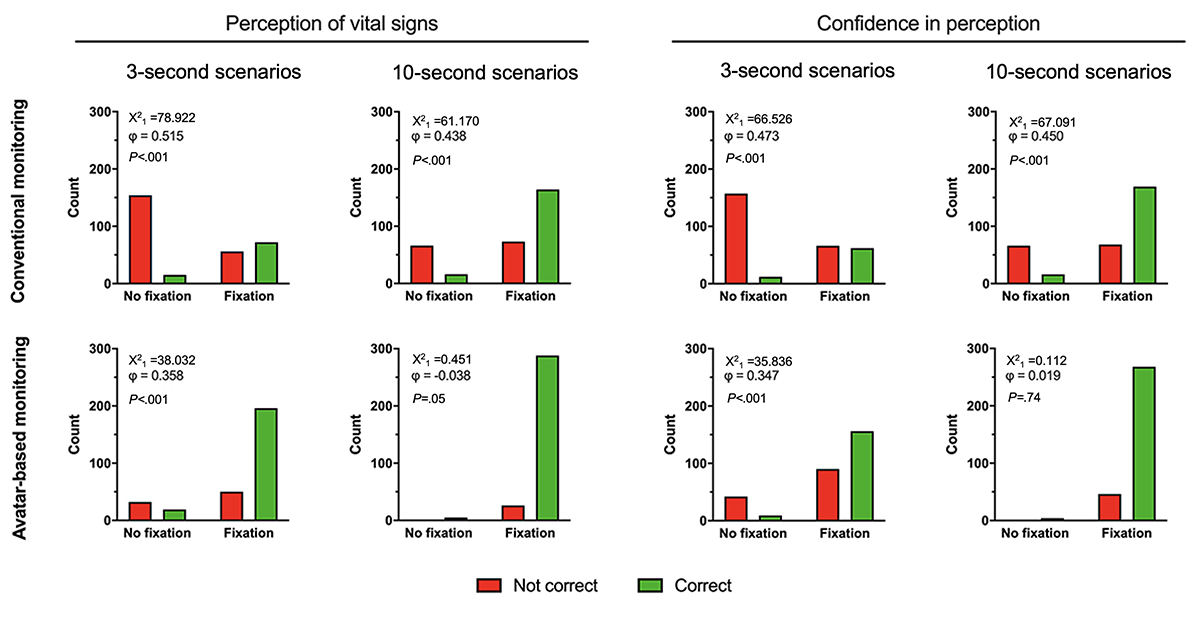
Cross tabulation bar graphs with chi-square tests for associations between visual fixation of a vital sign and the correct perception of the vital sign and the confidence in the correct perception.

A chi-square test for association was conducted between visual fixation of a vital sign and the correct perception of this vital sign. All expected cell frequencies were greater than five, except in the 10-second scenario with avatar-based monitoring. In this scenario, most participants were able to fixate on every vital sign and perceive it correctly. Accordingly, there was a statistically significant association between visual fixation of a vital sign and the correct perception of said vital sign in the 3- and 10-second scenarios with conventional monitoring (3-second scenario: χ^2^_1_=78.9; 10-second scenario χ^2^_1_=61.1, both *P*<.001) and in the 3-second scenario with the avatar-based monitoring (χ^2^_1_=38, *P*<.001). When significant, the association was moderately strong (Figure 4).

Similar results were achieved by a chi-square test for association between visual fixation of a vital sign and the participants’ confidence in having perceived it correctly (Figure 4).

## Discussion

### Overview

Patient monitoring is a central part of modern surgery, anesthesia, and intensive care [1,22]. Currently available monitors enhance perioperative safety [23,24]; however, they mainly show vital sign information as numbers and waveforms, which is not an ideal format for quick and easy interpretation [4,5]. An alternative monitoring technique, using an avatar-based representation of vital signs, has been found to enable anesthesiologists to grasp more vital sign information in a shorter time, resulting in improved diagnostic confidence and diminished perceived workload [8,16].

### Principal Findings

In this study, we evaluated eye-tracking data collected in two groups of anesthesiologists at two study centers. We recorded these data while the anesthesiologists were given the task to perceive vital sign information from patient monitoring scenarios presented in the two technologies (ie, conventional and avatar-based). Specifically, we evaluated how many vital signs and for how long these vital signs could have potentially been read by the participants according to the paths of their foveal or sharp vision across the screen. We found that participants were able to visually fixate more vital signs during the same time with avatar-based monitoring than with conventional patient monitoring. Nine of 11 vital signs were fixated more frequently per observation with avatar-based monitoring. Moreover, with avatar-based monitoring, participants fixated the vital signs for longer time intervals per recording, which might give them more time to process the information. More time to perceive the information may have been responsible for the reductions in perceived workload. In short, with the avatar, users see more information for a longer time. These findings were a consequence of the design of the avatar, with many of the vital signs spread out over a large part of the screen, and some visualized multiple times. For example, the vital sign “respiratory rate” can be interpreted by looking at the expiratory carbon dioxide “cloud” of the avatar and in the excursions of its lungs. The number of correctly perceived vital signs without a visual fixation accounted for less than 10% of the correctly perceived vital signs in all scenarios and with both technologies. This may have been influenced by the audio signal played in the conventional monitoring scenarios, which contained information on pulse rate and oxygen saturation. There may also have been some correct guesses without actual perception. In the avatar scenarios, some of the vital signs may have been perceived through peripheral vision, which we found to be an additional advantage of avatar-based monitoring [16].

The cases of visually fixated vital signs that were not correctly detected accounted for between 0% and 20% of all vital signs, depending on the scenario and technology, which might be explained by losses during processing after visual fixation, such as when a vital sign is forgotten or confused for another vital sign before being recalled. Numbers are glyphs that cannot be attributed solely to one vital sign; that is, it might be possible that a participant, although remembering the value of a number correctly, may misattribute the number to another vital sign with a similar range. Indeed, our data showed that when participants had to remember more than just a few vital signs in the more extended 10-second scenarios, the number of vital signs with a visual fixation that participants could not recall was more than twice as high in conventional monitoring than in avatar-based monitoring. These results correlate with research on the holding capacity of our working memory, which has shown that people can only store seven plus or minus two digits in short-term memory [25].

With avatar-based monitoring, nearly all participants were able to visually fixate and correctly perceive almost all the vital sign information in the longer (10-second) scenarios. This study shows the limitations of the single-sensor, single-indicator design of conventional patient monitoring, in which a single sensor on the patient feeds a single indicator on the patient monitor. The numbers must be individually read one after the other and then interpreted before meaning and a global mental picture of the situation can be derived [5,15]. With avatar-based monitoring, we found that the four critically important vital signs (pulse rate, blood pressure, oxygen saturation, and respiratory rate) remained perceptible for almost the entire duration of the monitoring scenarios. To perceive this same information, conventional monitoring requires four visual fixations, the eye movements in between them, and brainwork to interpret the meaning of the values. Avatar-based monitoring facilitates the interpretation work by the use of vital sign visualizations that have a logical commonality with the real phenomena they mirror and therefore do not require further mental translation by the user to be understood. The principle that a good model reflects the reality it represents is found both in principles of logic [14] and in situation awareness design principles, in which it is known as “presenting information directly” [4]. Anesthesiologists have mentioned information overload as a common problem in connection with patient monitoring [26]. In the future, more and more inexperienced users will likely monitor patients; therefore, ease of information transfer will be of paramount importance [27]. The ultimate benefit of Visual Patient should be an increase in patient safety. Although at this stage of its development we are not yet able to evaluate patient outcomes, the results of this and our previous studies fit into the context of situation awareness, decision making, and performance. Care providers must perceive and understand the available information before they can confidently make the correct decision and take the right measure [3,28]. Situation awareness failures have been identified as root causes of critical anesthesia events [29,30].

### Limitations

This study has some important limitations. For one, self-enrollment, based on interest in the technology, could have led to a selection bias. Less technology-savvy care providers may have achieved different results. Secondly, we recorded the source data in a simulated environment. The operating room and intensive care unit environment are very complex in real life; therefore, it is impossible to predict precisely how substantial the effects of an avatar-based monitor would be in these settings. However, it is plausible that effects would persist when used as a real patient monitor because the general physiological specifications of information intake do not change. A study with a high-end patient simulator in a realistic environment with the technology must be carried out as the next step of scientific evaluation on the way to producing a commercial product. Another potential limitation is the versatility of the eye-tracking method. Although we were able to validate the method through the positive correlation between visual fixation and correct perception, there are influences in perception that are not fully detectable by eye tracking, such as the influences of the audio signal, peripheral vision, and working memory. Particular strengths of this study include its multicenter design and the balanced enrollment of the different occupational groups and genders—a multivariate regression analysis rendered significant local effects, gender, profession, and scenario ordering effects unlikely. The within-subject study design minimizes the impact of interviewer variability of the eye-tracking method and other interparticipant variabilities [31,32]. The sample size adequately powered the analyses, and the significant magnitude and consistency of the differences observed between the two monitoring technologies increase the internal validity of the study.

### Limitations of Visual Patient Technology

Visual Patient has some inherent limitations. The technology simplifies vital signs into categories (ie, “too low,” “normal,” or “too high”). This preprocessing leads to improved intelligibility and diagnostic certainty but also reduces data accuracy (three discrete categories versus 300 different numbers in the case of pulse rate). Another limitation of the Visual Patient version used in this study is that it cannot yet display trends. This aspect is important because trend displays of conventional patient monitors can help care providers detect changes over time. In this context, it is important to note that we are developing avatar-based monitoring to improve information transfer, but not as a replacement of the conventional monitoring streams. Successful integration of the two technologies will be key for the success of Visual Patient, as with synthetic vision technology and numerical flight data.

### Conclusions

This study analyzed eye-tracking data to explain the improved information transfer with avatar-based patient monitoring. The avatar’s design, in which the vital sign information is presented as large-scale, integrated, colorful, and direct visualizations, allows users to see information about more vital signs with every glance and also see the vital sign information for a longer time with every glance at the monitor. In short, the way the avatar presents the information enables parallel perception of multiple vital signs at the same time, thereby increasing the number of visually fixated vital signs and the time available to view each vital sign. This study provides important groundwork for the future clinical validation of the concept. Future studies should examine the technology’s performance in simulator-based and then real-life studies.

## Appendix

Multimedia Appendix 1 Visual Patient educational video shown to the participants in this study.

Multimedia Appendix 2 Video demonstrating method used in study. Visual fixations longer than 50 milliseconds were analyzed and the vital signs within 2 centimeters of the fixation were counted. Therefore, the vital signs that participants could potentially have read during the recording (located within the potentially readable visual area) were identified.

Multimedia Appendix 3 Supplementary Figures 1 and 2, and Supplementary Table 1.

## Abbreviations

IQR: interquartile range
