# Supplementary material for: The Mechanisms Responsible for Improved Information Transfer in Avatar-Based Patient Monitoring: Multicenter Comparative Eye-Tracking Study
Source: J Med Internet Res. 2020 Mar 16;22(3):e15070. doi: 10.2196/15070 (PMC7105929; doi:10.2196/15070)
Supplement: Multimedia Appendix 3 [file jmir_v22i3e15070_app3.docx]

Supplementary appendix

*Supplementary Figure 1:* Flowchart of the study procedure. USZ=University hospital of Zurich (study center 1), KSW=Kantonsspital Winterthur (study center 2).


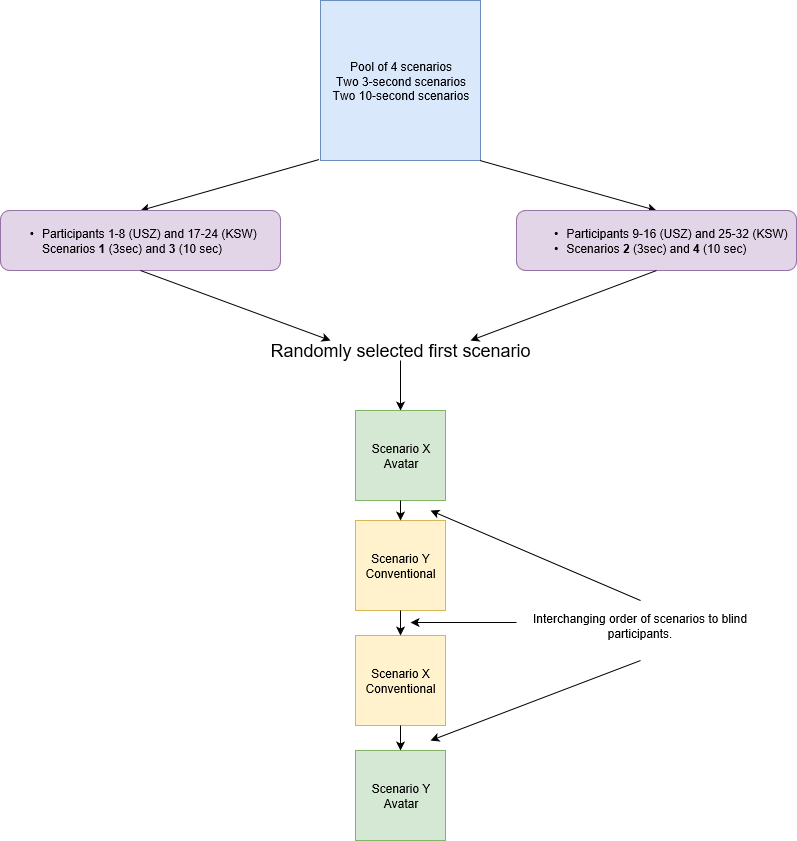


*Supplementary Figure 2:* Avatar-based monitoring compared to conventional patient monitoring: Median (with interquartile range) numbers of visual fixations for each vital sign, scenario, and technology. For example, participants in scenario 1 (3sec) with conventional patient monitoring had a median of 3 visual fixations recorded at a distance of 2 centimeters or less from a number or waveform corresponding to the vital sign pulse rate. In the same scenario with avatar-based monitoring the median number of visual fixations recorded at a distance of 2 centimeters or less of a surface of the avatar corresponding to pulse rate was 8. N for scenario 1 (3sec) was 12, for scenario 2 (10sec) 15, for scenarios 3 (3sec) 14 and for scenario 4 (10sec) 15.


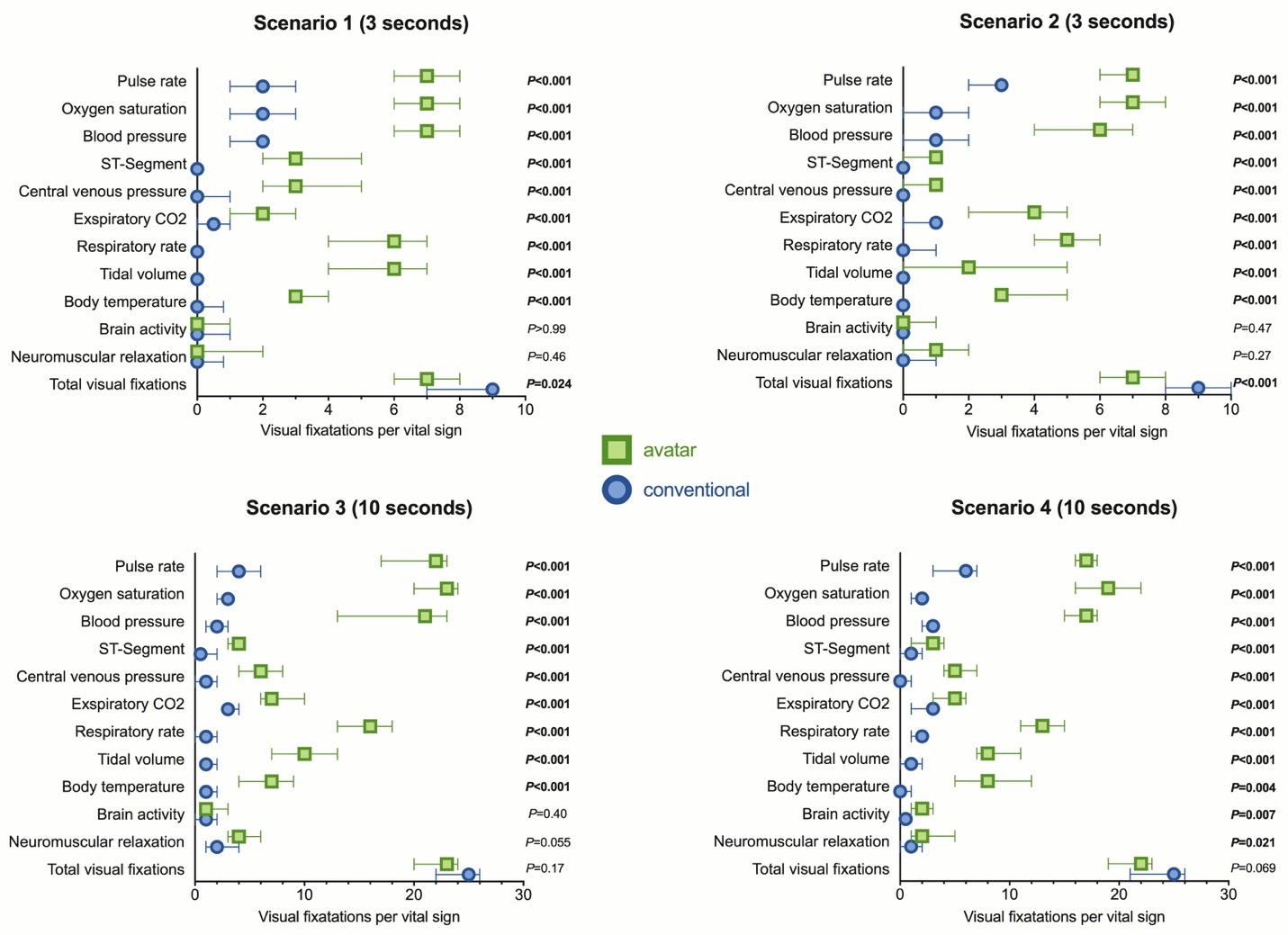


*Supplementary Table 1:* The visualisations and numerical values displayed in the four scenarios.

MmHg=millimetres of mercury, ml=millilitre, kPa=kilopascal. BIS=bispectral index system, SpO2=peripheral oxygen saturation, mV=millivolt, TOF=train of four.

|  | 1. Pulse rate  Visualisation  Condition  Numerical  value  (min-1) | 2. Arterial blood pressure  Visualisation  Condition  Numerical  value (MAP) [mmHg] | 3. Central venous pressure  Visualisation  condition  Numerical  value  (mmHg) | 4. Respiratory rate  Visualisation  condition  Numerical value  (min-1) | 5. Tidal volume  Visualisation  condition  Numerical value  (mL) | 6. Expiratory CO2 concentration  Visualisation  condition  Numerical value  (kPa) | 7. Body temperature  Visualisation  condition  Numerical value  (°C) | 8. Brain activity  Visualisation  condition  Numerical value  (BIS) | 9. Peripheral oxygen saturation  Visualisation  condition  Numerical value  (%SpO2) | 10. ECG ST-Segment  Visualisation  condition  Numerical  value  (mv) | 11. Neuromuscular relaxation  Visualisation  condition  Numerical  value  (%TOF) |
| --- | --- | --- | --- | --- | --- | --- | --- | --- | --- | --- | --- |
| Scenario 1  (3 sec) | safe  69 | safe  117/70 (86) | too high  21 | safe  15 | safe  543 | safe  4.5 | safe  36.8 | low  24 | too low  67% | safe  0.1 | no relaxation  100 |
| Scenario 2  (3 sec) | too high  261 | Safe  125/76 (92) | too low  1 | safe  13 | safe  410 | too high  9.3 | too low  33.4 | high  99 | too low  77 | safe  0.1 | relaxed  26 |
| Scenario 3  (10 sec) | too high  257 | too high  247/173 (197) | safe  8 | safe  11 | safe  552 | too high  12.7 | safe  37.2 | low  47 | too low  74 | abnormal  -0.9 | relaxed  41 |
| Scenario 4  (10 sec) | too low  13 | too low  36/5 (14) | safe  8 | safe  12 | safe  452 | safe  4.4 | too high  41.4 | high  96 | safe  96 | safe  0.0 | no relaxation  94 |
